# A Digital Health Service for Elderly People with Balance Disorders and Risk of Falling: A Design Science Approach

**DOI:** 10.3390/ijerph19031855

**Published:** 2022-02-07

**Authors:** Andréa Gomes Martins Gaspar, Luís Velez Lapão

**Affiliations:** 1 Instituto de Higiene e Medicina Tropical (IHMT), Universidade NOVA de Lisboa (UNL), 1349-008 Lisbon, Portugal; luis.lapao@ihmt.unl.pt; 2 Hospital Beatriz Ângelo, 2674-514 Lisbon, Portugal

**Keywords:** balance disorders, falls, healthy ageing, elderly care, eHealth, digital health

## Abstract

In this study, a design science research methodology was used aiming at designing, implementing and evaluating a digital health service to complement the provision of healthcare for elderly people with balance disorders and risk of falling. An explanatory sequential mixed methods study allowed to identify and explore the dissatisfaction with electronic medical records and the opportunity for using digital health solutions. The suggested recommendations helped to elaborate and develop “BALANCE”, a digital service implemented on the METHIS platform, which was recently validated for remote monitoring of chronic patients in primary healthcare. “BALANCE” provides clinical and interactive data, questionnaire pre and post-balance rehabilitation, tutorial videos with balance exercises and patient-recorded videos of the exercises. This digital service was demonstrated, including five elderly patients with clinical recommendations for balance rehabilitation at home. Finally, the authors conducted two focus groups with the participants and their caregivers as well as with physicians. The focus groups aimed at exploring their satisfaction level, needs of adjustment in the “BALANCE” service and strategies for applicability. The digital healthcare service evaluation revealed a significant potential for clinical applicability of this digital solution for elderly people with balance disorders and risk of falling.

## 1. Introduction

**Figure 1:**
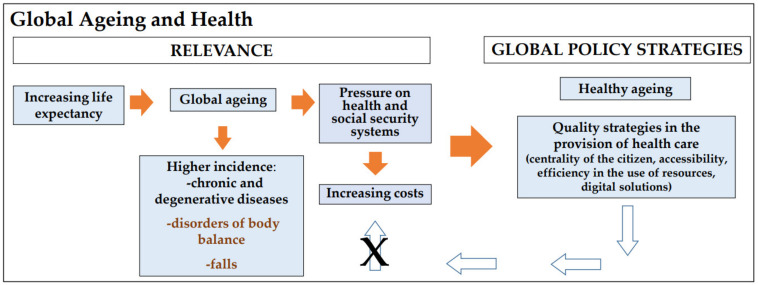
Strategies for a sustainable ageing (Authors’ own elaboration).

In most countries, the life expectancy growth rate has not been met by an increase in healthy life years [1,2,3,4,5]. With ageing, a growing prevalence of multiple comorbidities, chronic diseases, balance disorders and falls affecting the independence of the elderly has been observed [6,7,8,9,10,11]. However, healthy ageing does not mean disease-free. Even in the presence of disease, healthy ageing means living well with the disease under control and with functional ability, which is seldom observed [3]. The dearth of healthy ageing among the elderly has led to inappropriate use of physical and human resources, to multiple medical visits, diagnosis tests and treatments [11,12,13]. The ageing process has challenged the sustainability of the healthcare systems, including the Portuguese health system [1,5]. To address this, several approaches have been suggested, such as person-centered health systems and use of digital devices [2,3,14,15,16,17,18] (Figure 1).

Several studies have explored the potential of digital health solutions to assess and monitor the elderly with balance disorders and risk of falling. The heterogeneity of populations, methodologies of study, eHealth devices and services as well as time of follow-up have not allowed for clear comparisons. Therefore, the actual clinical applicability of these solutions still needs to be further explored [19,20,21]. In this study, we aimed to design, develop, implement and evaluate the use of a digital health service for the complementary provision of care for the elderly with balance disorders and risk of falling.

## 2. Materials and Methods

### 2.1. Study Design and Materials

**Figure 2:**
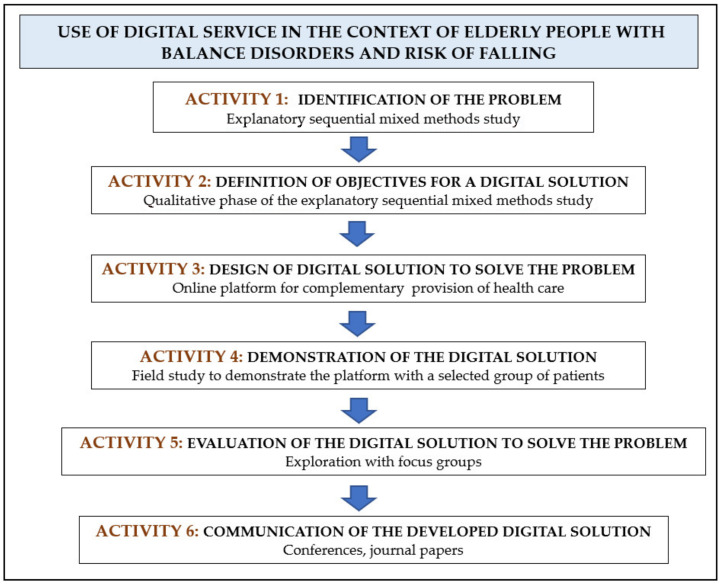
DSRM activities and tasks. Activities 1 and 2 were already published [23].

Using Design Science Research Methodology (DSRM) [22], a digital service for the remote management and monitoring of elderly people with balance disorders and risk of falling was designed, implemented, demonstrated and evaluated (Figure 2).

An explanatory sequential mixed methods study [23] was previously performed. This study identified the clinical practice difficulties, the relevance of using digital solutions and the strategic options to develop a digital service targeting this profile of patients (Activities 1 and 2 of the DSRM). First, we elaborated and adapted an open questionnaire with 18 multiple choice questions, available through the website of the General Medical Council of Portugal (Activity 1). Second, one of the authors conducted semi-structured interviews for better understanding of the results obtained from the quantitative study and to explore how to develop an efficient digital service to support healthcare (Activity 2). The eligible participants were specialist physicians focused on healthcare provision in Portugal for the elderly with balance disorders and risk of falling [23].

Based on the results obtained in the previous mixed methods study, especially the recommendations, the “BALANCE” digital service was conceived and designed, as an online service to provide balance care services (Activity 3). The purpose of the digital service had been defined to provide complementary care for elderly people with balance disorders and risk of falling, allowing a closer physician–patient interaction and encouraging a more active participation of the patients and caregivers. From August to September 2020, we designed an online service, called “BALANCE”, following the otoneurologic clinical approach of patients with balance disorders [24]: clinical data, clinical examination findings, instrumental test findings, treatment and the Dizziness Handicap Inventory (DHI) questionnaire [25], a validated self-reported questionnaire that quantifies the impact of dizziness on daily life, pre and post- rehabilitation program. From October 2020 to April 2021, “BALANCE” digital service was implemented and integrated into the digital platform, METHIS (Multimorbidity Management Health Information System) [26], a web-based platform with three relational databases using PostgreSQ and fully integrated with Zoom (Zoom Inc.). The platform METHIS was recently validated for providing remote monitoring services for chronic patients in primary healthcare [26]. For better patient guidance, eight tutorial videos were recorded by the first author (consultant physician), performing the specific balance exercises. The videos were uploaded to the YouTube^®^ platform as unlisted with restricted access, and their links were integrated to “BALANCE”. Finally, until July 2021, the “BALANCE” digital service was adjusted to requirements. One otorhinolaryngologist, two experts in digital platforms and one professor of health information systems collaborated in the development and implementation of this digital service.

To demonstrate the “BALANCE” digital service (Activity 4 of DSRM), we performed a proof-of-concept study [27] from August to November 2021. A set of patients was followed at the Otorhinolaryngology and Head and Neck Surgery Service of Hospital Beatriz Ângelo (HBA). They were invited to participate, respecting the following inclusion criteria: (a) age of 65 years or older; (b) complaints of clinically decompensated balance disorder with risk of falling, confirmed with objective clinical examination findings; (c) instrumental tests performed to assess inner ear function, such as videonystagmography or cervical vestibular evoked myogenic potentials, allowing for prescribing balance exercises according to the clinical condition; and (d) balance rehabilitation indication for patient with clinically decompensated balance disorder; each participant performed vestibular rehabilitation at HBA, which allowed the assessment of the patient’s ability to walk without support and he/she would have the capacity to perform the balance exercises at home. We excluded patients with the following conditions: (a) neurological pathology diagnosis at the time of the inclusion in the study, such as a neurodegenerative disease; (b) ophthalmological pathology diagnosis with severe visual acuity that did not allow one to see the computer screen; (c) osteoarticular pathology diagnosis with reduced mobility of the lower limbs; (d) a clinical condition that did not allow the regular performance of physical exercises, such as decompensated cardiovascular pathology or acute infection; (e) a cognitive alteration according to the Mini-Mental State Examination (MMSE), using operational “cut” values for the Portuguese population, elderly individuals with less than 22 points for 0 to 2 years of education, less than 24 points for 3 to 6 years of education and less than 27 points for participants with education equal to or greater than 7 years were all excluded [28]; (f) insufficient comprehensive to understand the study; and g) limited access to the internet. All selected participants provided an email address for later access to the digital platform and to receive a automatically-generated password. The patients and caregivers also received individual instructions by the consultant physician, via a Zoom teleconsultation, on how to access and operate the platform.

After the proof-of-concept study, we conducted two focus groups [29] using the Zoom platform (due to the SARS-CoV-2 pandemic limitations) to discuss and explore the benefits, constraints, adjustment requirements, new strategies for clinical applicability of the “BALANCE” service and the satisfaction level (Activity 5 of the DSRM). On 28 November 2021, the main researcher conducted a focus group with the participants of the proof-of-concept study and their caregivers, that is, the proof-of-concept users of the “BALANCE” digital service. Five primary thematic categories were discussed: benefits and constraints of the “BALANCE” service, satisfaction, strategies suggested and interest in continuing to use the platform (Appendix A). The link, identity number and password of the meeting were sent to the participants, via an asynchronous message system provided by the digital platform. On 5 December 2021, the same researcher conducted a focus group with physician experts in healthcare provision for elderly people with balance disorders and risk of falling. The sample was intentional [29], with a purposeful search for physicians with the following characteristics: (a) providing care for the elderly with balance disorders and risk of falling; (b) coordinating function in public health units; and (c) being easily accessible to the interviewer. The potential participants were invited by the interviewer, in person or via mobile phone. All physicians received a specific message, via multimedia messaging service (MMS), WhatsApp or email, with information about the study and audio recording, link, an identification number and password for the Zoom meeting. Before the discussion was initiated, the researcher showed a summary of the project with the main results of the various activities carried out and showed the functionalities of “BALANCE”. Six thematic categories were covered: benefits and constraints of “BALANCE” digital service, satisfaction, recommended adjustments for the “BALANCE” digital service, proper clinical applicability and interest in this digital care service (Appendix A).

Finally, we disseminated the results of these activities in oral communications, posters and papers published in conference proceedings (Activity 6). Two papers were already published in peer reviewed journals [21,23].

### 2.2. Data Analysis

We performed a descriptive analysis of demographic data of all participants in the different activities [29].

The information of the interviews and focus groups was manually coded and transcribed by the interviewer, allowing for content analysis to be carried out [29]. We entered words in round brackets, enabling a better comprehension of the quotes.

### 2.3. Ethical Considerations

The participants of the proof-of-concept study received a copy of the signed consent form referring to the participation in the demonstration study and in the focus group on the satisfaction of the digital solution. The patients also signed a document pledging not to share or publicize the videos nor to record the videos available in the study.

The interviewees and the participants of the focus groups were informed that they could leave the study up until one month after the interview or Zoom meeting date. At any time, the patients could withdraw from using the digital platform. Regardless of their participation in the study, all of them were followed up with on the face-to-face consultation at HBA.

Audio of interviews was recorded upon authorization from the participants. In both focus groups, all the participants received an automatic message authorizing the meeting’s recording, from the Zoom service, after accessing the link to the meeting. The researcher asked participants to turn off the camera during the recording of the Zoom meeting, since the objective was only to collect the audio.

All the information was treated confidentially. The interviews and meetings were manually coded and transcribed by the interviewer for content analysis. The transcriptions omitted information to avoid identifying participants. All data were kept anonymous [29]. The information and audio records were kept in a safe place (external disk with access code) within the period provided by the Portuguese law [30] and General Data Protection Regulation (GDPR) [31], safeguarding the confidentiality of the information obtained at all times.

The participant engagement in the activities was voluntary.

## 3. Results

Firstly, the previous mixed methods study confirmed a dissatisfaction with the current medical data relative to the elderly with balance disorders and risk of falling as well as the dissatisfaction with lack of system interoperability, which led to multiple emergency visits and uncoordinated diagnostics and treatments. We also verified there was an opportunity to explore a digital solution in this context, as most physicians considered this strategy relevant [23]. The recommendations from the interviews (e.g., inclusion of clinical data recorded by either the patient or the caregiver, interactive questionnaires and calendars on care episodes and triggering factors for deterioration of balance disorders and falls, availability of tutorial videos with balance exercises and possibility of uploading patient videos for clinical follow-up) helped to define the objectives and design the “BALANCE” digital service for a complementary provision of healthcare.

### 3.1. Design of a Digital Solution to Solve the Problem

The results of the design of “BALANCE” digital service were a set of functionalities following both medical and patient requirements enabled to define both medical and patient profiles:

(a) Quick access to balance deterioration or recent fall data;
(b) Provide tutorial videos with specific balance exercises (1. Walk; 2. Walk turning the head to the right and to the left; 3. Walk lifting your legs; 4. Walk lifting the legs and turning the head to the right and to the left; 5. Walk with legs progressively apart. Video A—Moving target with motionless head; Video B—Moving head with motionless target; Video C—Moving head and target);
(c) Regular monitoring and adjustment of vestibular exercises.

#### 3.1.1. Medical Profile

The physician could register the patient data on the platform and create an individual clinical record. By clicking on “BALANCE” service, the following set of six functionalities are available:

(a) Balance monitoring (i.e., access to clinical data): the SOAP framework [32] was used, with a checklist and free-text, with the following: Subjective (complaints: vertigo, unsteadiness, difficulty walking at home or outside, need for crutches or canes to walk, falls, triggers, hearing loss or tinnitus); Objective clinical examination findings (gait, otoscopy, clinical and instrumental examination, Time Up and Go Test (TUGT), Berg Balance Scale (BBS), Tinetti Performance e-Oriented Mobility Assessment (POMA), cochleovestibular instrumental exams, imaging exams and blood tests); Assessment based on the information collected in the previous two sections, with the summary of the salient points; Plan (treatment: medication and balance rehabilitation);
(b) Patient balance deterioration records: the physician could identify an eventual clinical deterioration and triggers, with a checklist and free-text structure data recorded by the patient from home;
(c) Patient’s fall records: the physician could be alerted of an eventual episode of falling, triggers and consequences, with a checklist and free-text data recorded by the patient;
(d) Dizziness Handicap Inventory (DHI) [25]: the physician could access the 25-item questionnaire and the final score, automatically summed;
(e) Balance rehabilitation prescription: the physician could also provide additional information with free-text;
(f) Patient record about exercises performed: the physician could monitor the daily frequency of the balance exercises performed and access a possible video link uploaded by the patient with exercises performed.

#### 3.1.2. Patient Profile

After the registration on the platform by the physician, the patient received, via email, an automatic individual password (unknown to the physician). After logging in, the participant was able to forward messages to the assistant physician and access “BALANCE”. By clicking on this, the following set of five functionalities become available:

(a) Patient balance deterioration records: the patient or caregiver could record a recent deterioration of the balance. This information, either with checklist and free-text, can also be visible in the medical profile;
(b) Patient’s fall records: the patient or caregiver can record the episodes of falling; this information, checklist and free-text, can be also visible in the medical profile;
(c) DHI questionnaire [25]: to be completed before and after the balance rehabilitation program; the final score can be automatically summed and is visible in the medical profile;
(d) Recorded tutorial videos with balance exercises: each one presents instructions on the exercise to be performed, allowing for viewing in slow motion and at normal speed of the exercise;
(e) Patient record about exercises performed with checklist structure: the patient can upload a recorded video link of other exercises performed. This information can also be visible in the medical profile. At any time, both physician and participants can send or receive messages with questions or comments, enabling a more dynamic physician–patient interaction. All data are processed with security, respecting the General Data Protection Regulation rules.

### 3.2. Demonstration of the Digital Solution

#### 3.2.1. Participants

Since we used a proof-of-concept approach as an initial intervention to demonstrate the “BALANCE” service, only a small set of participants was included: five female patients, aged 70–83 years old, with a regular balance follow-up at Hospital Beatriz Ângelo (HBA), Lisbon, Portugal (Table 1). One participant, a male patient aged 86 years old, withdrew from the study, stating that he depended on his daughter’s support and that she no longer had the time to access the platform. In fact, no data was filled on the platform about this participant. However, the patient had been followed at HBA, with face-to-face consultation, as were the other participants.

All the patients reported initial complaints about clinically decompensated balance disorders and instrumental exams performed for the evaluation of inner ear function. Additionally, three patients usually used nearby furniture as support to maintain balance during gait. One participant often needed this support and another used it, mainly in low-light environments or with uneven floors to prevent falls. They had already completed a rehabilitation program at HBA and had indicated they were carrying out balance rehabilitation at home. None of them were revealed to have a cognitive disorder according to the Mini-Mental State Examination (MMSE), considering the operational “cut” values for the Portuguese population [28]. Participant 1, after inclusion in the study, was evaluated for suspected Lewi Body disease. However, her MMSE score was 27/30, hence the reason why the researchers chose to keep her in the study.

#### 3.2.2. Remote Monitoring

**Figure 3:**
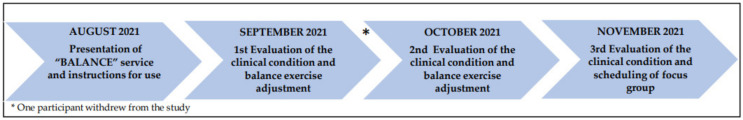
Description of the remote contact schedule.

On a daily basis, each patient was remotely monitored for clinical conditions. The physician contacted each of them at least four times during the study (Figure 3).

In the first step of the study, the patients (or the caregivers) were individually trained on how to access (via a secure and unique link) and use the “BALANCE” service. This training was performed via Zoom due to the SARS-CoV-2 pandemic limitations. During the screen sharing, all the functionalities, including the videos with balance exercises, were shown and an email with instructions about exercise frequency was sent. According to the clinical condition and limitation, each patient received personalized instructions about the balance exercises. We also asked them to complete the pre-questionnaire (DHI). About a month later, we called each patient by mobile phone, to establish how his clinical condition was and the constraints with balance exercises. Accordingly, we adjusted the balance rehabilitation schema, including more or fewer exercises. The instructions were sent by email. The second individual evaluation, both via Zoom and available on the platform (for future reference), allowed us to re-evaluate the clinical condition and re-adjust the balance exercises. Finally, during November 2021, we re-evaluated the clinical condition and asked the patients and caregivers, via mobile phone, to complete the post-questionnaire (DHI). We also invited them to participate in the focus group.

#### 3.2.3. Participant and Caregiver Registration in the Digital Platform “BALANCE”

Despite vertigo complaints by four of the patients, only two caregivers actually registered this information on the platform (Table 2).

These records motivated an additional medical contact with the patients, via mobile phone, to better evaluate their conditions and to intervene as early as possible. No fall was recorded during this period. Only two caregivers registered and reported the exercises being performed for less than a month. However, they sent emails about their parents’ limitations with balance exercises, expecting some support from the interaction that is possible through the digital platform. The patient with the elderly caregiver had no record registered on the platform. No patient or caregiver sent an individual video with exercises performed, as requested by the consultant physician. Nevertheless, the comparison between pre and post DHI score already revealed some benefits of the balance exercises. This information was sent, via email, as positive feedback to participants, motivating them to continue performing the balance exercises.

### 3.3. Evaluation of the Digital Solution to Solve the Problem

#### 3.3.1. Focus Group Socio-Demographic Participant Data

A researcher (and first author) conducted a focus group with five patients and five caregivers, all involved in the proof-of-concept study. This information is available in Table 1.

A week later, the same researcher conducted the other focus group with eight physicians who provided healthcare to the elderly in Portugal, having different technology knowledge and experience. Seven participants had coordination positions or were ex-coordinators of their own specialty in their health units, and one was an ex-coordinator of a medical residency in a public health unit in Portugal (Table 3).

#### 3.3.2. Focus group-Content Analysis

In the focus group with the participants of the proof-of-concept study, five primary thematic categories were discussed. From the data analysis, 11 subthemes emerged. Additionally, six other thematic categories were discussed in the focus group with physicians. From data analysis, 15 subthemes emerged (Table 4).

All physicians recognized the increased importance of telemedicine, especially in recent days: *The* (SARS-CoV-2) *pandemic has changed many things … therefore, we have to look for new solutions* (Physician 7); *… the pandemic brought bad things but it also brought good things, namely in telemedicine* (Physician 4); *It might be an opportunity for eHealth*… (Physician 5); *The new tools* (telemedicine) *allow you to reach more people* (Physician 1); *It*… (telemedicine) *requires us… to review everything* (clinical file) *and with that we are even more prepared* (with more quality of data) *to the consultation* (Physician 5); *There is no doubt that Portugal is ageing badly*… (so) *we have many people who would benefit … and caregivers can (also) benefit in the future* (as they are getting older) (Physician 3).

In fact, both groups of interviewees mentioned many benefits from using the “BALANCE” digital service. They pointed out the patient comfort: *I had to go* (to the hospital) *by public transport or my daughter would take me* (to the hospital) *most of the time. And so* (with the platform), *it is not necessary* (Patient 2); *It increases the comfort… the safety of the elderly …*(Physician 2). The benefit of closer physician–patient interaction was also mentioned in both focus groups: *I think it was the best…the interaction with the physician… and really the physician was able to answer almost immediately…* (Caregiver: Patient 5); *…to keep therapy* (balance rehabilitation) *at home, with all this help, it is fantastic* (Physician 8); *It increases… the connection with the health professional* (Physician 2); *I am fully convinced that, with this type of strategy, we can help our patients … Basically, what they (patients) need is a close and frequent monitoring … physician available … to guide them through their difficulties* (Physician 5). The patients and caregivers also highlighted the medical involvement: *The physician* (i.e., the researcher) *is a person very concerned about us… in the other day, what I had was not even related to balance disorder…and the physician called anyway, worried to know if I was alright* (Patient 2); *… really the physician answered almost immediately* (Caregiver: Patient 5). The availability of tutorial videos with balance exercises was highly praised: *The* (recorded videos) *are very good… They help to remember* (the exercises) (Caregiver: Patient 5). This availability of videos also allowed caregivers to understand the exercises and create strategies to make their family members less dependent: *My mother watched the videos. To avoid forgetting, we wrote down what should be done…* (Caregiver: Patient 4); *The videos motivated the realization of exercises outside the home, even in an unstable surface. I memorized most of them…even when I was walking on the street…I was doing them* (the exercises) *… normal path or in the sand. It was amazing. I think this gave me greater security* (Patient 2)*;… she* (the mother) *was even doing* (balance exercises) *in the market…* (Caregiver: Patient 4). The potential lower use of face-to-face resources was also mentioned: *Perhaps it* (digital solution) *will consume less face-to-face resources, with some people proactivity*. (Physician 1); *I think it is interesting … the patients can share their own difficulties and abilities through a mobile phone, a webcam…* (Physician 8). Moreover, the interviewees highlighted the potential for better patient motivation and adhesion to the therapy: *I want to thank. I think this* (digital platform) *is facilitating, even though it may seem like an obligation* (doing the exercises), *it encourages and serves even for us* (caregivers)*,… as we are going there* (we are getting older) (Caregiver: Patient 3); *Strategies like this can allow … greater adherence of patients to therapy* (Physician 3)*;…if they* (patients) *are at home and they have the perception that Andréa Gaspar* (the name of the reseacher, for example) *is able to assist them, to give recommendations, to know what difficulties they were facing… they become more interested… even if Andréa Gaspar, or whoever, is doing the* (remote) *monitoring and asks how many hours* (the patient) *has spent on the couch, they will probably spend fewer hours on the couch and practice the* (balance) *exercises recommended than if they do not have any support or if they have support from caregivers who usually do not know how to provide this type of support* (Physician 8). In fact, the importance of carrying out balance exercises was recognized: *I think it’s important to do* (balance exercises)… *sometimes doing more…sometimes doing less* (Caregiver: Patient 4).

However, some of the interviewees pointed out possible constraints in both focus groups, for example the user profile definitions: *People reached presbyvertigo* (prebivestibulopathy) *sooner than digital resources arrived. There are technical difficulties for this* (use of telemedicine by the elderly) (Physician 8); *We can use* (digital solution) *only with patients, with some differentiation. I think this is the big issue with this tool* (Physician 7). However, this point of view was not held by all physicians: *It is a mistake for us to think that elderly are completely averse and incompetent to use electronic resources. I think that these people are sometimes available to experiment…especially when they use it for their interest… namely, in their own health.* (Physician 5); *There are many people who can benefit from this project with the proper training of their caregivers, even those who consider themselves info-excluded* (Physician 1). In fact, all participating caregivers recognized the existence of their elderly family members’ limitations on the use of “BALANCE” digital service: *My mom couldn’t do that* (use the digital solution) … (Caregiver: Patient 5); *Of course, I have no difficulty at using the* (digital) *platform… But to apply it to elderly people, … it’s not easy* (Caregiver: Patient 3). About this, Patient 1 mentioned: *I have been doing* (the exercises) *according to what the physician explained to me* (all participants received, via email, individual instructions with the recommended exercises and videos). *I never saw the videos* (during balance rehabilitation). After sharing the computer screen, her caregiver, who is her 72-year-old husband, said: *Yes, the physician* (the researcher) *has already presented this to me. She* (the physician) *already has presented it at the beginning* (in the first meeting, via Zoom, to explain how to use of the digital platform)*… … I simply forgot about it*. The caregivers of patients 3 and 4 also highlighted their difficulty with using “BALANCE” digital service on the mobile phone: *On the computer it’s easier* (the use of the digital solution) *than on the mobile phone*. The lack of time to record the balance exercises performed was another constraint pointed out: *…the daily record of exercises… we do not always have the computer on …* (Caregiver: Patient 3). The potential resistance of healthcare professionals as a constraint for clinical applicability of the “BALANCE” digital service was also mentioned: *…there will be some difficulties in terms of human resources…* (Participant 4); *It will always cost in the beginning of the process, when there is something new, there is always some resistance…* (Physician 7); *I often hear… that consultation of Otorhinolaryngology has to be a face-to-face consultation at all times… I think this is not true… the literature and articles…they have already demonstrated that it is not true… I know there is great resistance to change… It is necessary to have people … to demonstrate that we have to accept other alternatives and that they are useful* (Physician 5). The physicians also considered the lack of time as another barrier for clinical applicability of this digital solution: *It* (the use of this digital platform) *is an advantage that… must be compensated with specific time for this… my doubts are what time we* (physicians) *have to do this* (remote) *monitoring… …time must be allocated for this purpose* (Physician 5).

Despite not having seen the available tutorial videos, Patient 1 was motivated and performed the exercises according to email instructions and rated the remote physician–patient interaction 20 out of 20 values (on a scale from 0 to 20 points, 20 points for the best rating of level of satisfaction). Relative to the other participants of the proof-of-concept study, all caregivers rated the digital platform with 20 points, except for the caregiver of Patient 4. She rated it with 19 points, recommending specific adjustment on the available services: *There are* (in the digital platform) *items that we don’t use and then it gets a little more confusing*. (Caregiver: Patient 4). In fact, these “items” were part of the METHIS platform, related to the follow-up of patients with chronic pathology in primary healthcare. Another suggestions pointed out was the recording of the videos without protective equipment: *I think that, in the future, if it is possible to record without the protective equipment it would help more to be able to see the whole face… in the* (videos of) *walking, it would also be possible to better identify* (the exercises) (Caregiver: Patient 4). Actually, these videos were recorded with the researcher using individual protective equipament due to the SARS-CoV-2 pandemic. The recording of balance exercises performed was another point to be changed, avoiding the daily recording of exercises performed. When asked if there was a need to add more functionalities in the “BALANCE” *digital* service, the patients and caregivers did not agree: *It* (the digital platform) *couldn’t be much more complex* (to use)…(Caregiver: Patient 5). When asked whether puppet-animated videos could be used, patients and caregivers stated that recorded videos should only include people as models, such as the recorded videos added on the platform.

Regarding medical satisfaction on using the platform, only Physician 6 revealed some dissatisfaction with the digital service: *It is not clear enough in the information provided that the systems allows the physician to assess the patient’s evolution*. He suggested the adjustment of the “BALANCE” digital service with the inclusion of automatic tools: *… to analyze the videos* (sent by patients)*… creating alerts… so that the physician can intervene and make corrections in the patient’s rehabilitation process*. Other strategies for a better clinical applicability were discussed. For example, the restructuring of working hours were discussed: *Everything is possible, as long as there is a willingness…* (Physician 7);*… make the activity* (of the digital solution) *accountable as medical production* (within working hours)*… so that physicians and other professionals could have time for these activities* (Physician 5); *It is important to integrate into the schedule* (of health professionals)*, it is not beyond the schedule… If not, it is something* that *becomes a volunteer and the volunteer is temporary* (Physician 2). The use of the digital solution by other health professionals was also suggested: *If we can generalize this tool* (digital solution), *not only with the support of caregivers but also with the support of nurses…* (Physician 1). The interoperability was also remembered as an issue: *this is a tool that we can use, as long as the interoperability with other tools is guaranteed …* (Physician 1). They also discussed the importance of funding and continuity of digital solutions not only at the regional level but also at the national level, with the recognition of the hierarchy: *I will try to give you my experience… from two studies* (with telemedicine) *… I tried to keep elderly people at home with a better quality of life and they were not institutionalized. The biggest problem…. It was its end…I think the elderly have already lost a lot… in their lives. And when we give them a better quality of life, when we increase their ability to be at home… when we provide better caregivers* (with training of caregivers)*… we can’t have an end* (of the study). *I think that…they* (projects) *have to include more Ministries to be more sustainable…* (Physician 2).

Physician 6 also highlighted the importance of the involvement and integration of several professionals in designing and implementing digital solutions: *… these things only gain with exchange between the various centers…because we all learn together.*

All the physicians considered the digital platform as interesting and agreed with the potential of the digital solution as a complementary healthcare provision: *This project is interesting and it can move forward… and no longer just a study* (Physician 6); *There is undoubtedly a large percentage of patients who could benefit* (from the “BALANCE” digital service) (Physician 3).

All patients expressed their willingness to continue using the digital platform. One participant justified: *And if we* (patients), *from time to time, need to see the videos* (with the balance exercises) … *it’s better* (Patient 2).

## 4. Discussion

The systematic review [21] previously identified the lack of valid guidance of digital devices for a better clinical provision of healthcare in the context of elderly people with balance disorders and risk of falling [19,20]. Thus, to address and study this issue, DSRM [22] was used to design and integrate the “BALANCE” digital service to the platform METHIS [29], as suggested by strategies previously discussed in the explanatory sequential mixed methods study [23]. The “BALANCE” digital service was demonstrated and evaluated with a set of patients. We verified that there is an important potential for clinical applicability of this digital solution for the elderly with balance disorders and risk of falling.

The identification and exploration of the digital medical constraints relative to healthcare provision in this context, and the recommended strategies allowed us to elaborate the “BALANCE” digital care service. This digital solution allowed to: (a) upload and access clinical data, in checklist format and free-text structure, according to the SOAP model; (b) upload and access interactive data, in checklist format and free-text structure, allowing closer physician–patient interaction, complementary remote monitoring, early detection of clinical deterioration and adjustment of recommended balance exercises; (c) access the final score of the DHI questionnaire completed by the patient and compare the results before and after carrying out the balance exercises; (d) access tutorial videos with balance exercises in slow motion and at normal speed, for a better patient orientation. No video recorded demonstrating patients’ performances while carrying out the exercise was uploaded onto the platform, which could not have allowed for the physician’s “real-time” assessment.

Both participants and caregivers’ engagement in the demonstration of the “BALANCE” digital service was verified, as well as encouraging their elderly family members and teaching them how to access the exercise videos. Despite the 72-year-old caregiver not having accessed the videos as instructed, the patient was carrying out the exercises according to the medical remote interaction (Figure 3). This revealed the motivation of this patient and the limitation of the use of digital technology by an elderly patient and elderly caregiver, which had already been discussed in both focus groups. Instead of using the messaging functionality via the “BALANCE” digital service, this caregiver requested the physician’s email as a form of rapid contact, revealing the need for more intense digital training to use the platform. The patients and caregivers highlighted the medical involvement with quick response to their questions. In fact, the involvement of patients, caregivers and healthcare professionals is one key point for the success of digital interactive solutions [33,34].

Using focus groups, we explored the potential contributions, constraints for clinical applicability, satisfaction and strategies to improve the digital solution. In fact, digital health end-user experience is important, listening and engaging with patient, family members, caregivers, health professionals and policymakers to improve the implementation of digital solutions in clinical contexts [3,34,35].

As observed in other studies [14,15,17,36], the participants of the two focus groups highlighted several benefits of a digital rehabilitation service: comfort, closer physician–patient interaction anywhere, the potential for patient’s motivation and engagement with self-care. Additionally, the availability of the tutorial videos with balance exercises was considered as a positive factor, allowing patients to remember the exercises and motivating them to exercise, including outside the home. These videos also allowed caregivers to understand the recommended exercises. In fact, the advantages of internet based balance rehabilitation with videos had been already pointed out [36]. However, we also performed remote daily monitoring, allowing for additional physician–patient interaction in case of uploaded data regarding clinical deterioration.

However, according to the literature [37,38,39,40,41], some remaining constraints for the clinical applicability of digital devices have been discussed: limitations of elderly people in accessing and using technologies, need for previous digital training and caregiver support. In fact, the caregivers’ deep involvement and training were essential in our study as was reported in previous research [33,34].

Unfortunately, in our study, even the caregivers had difficulty in using the “BALANCE” digital service on the mobile phone support, pointing to the need for future adjustments. The digital devices must be adequate to match the abilities of this population as highlighted by other researchers [33,38,42].

The caregiver’s unavailability to upload daily data about exercises performed is another issue that needs to be considered. The suggested inclusion of automatic tools to objectively analyze patient’s videos may add value (and usability) to the platform “BALANCE”.

As already identified [5,33,37], the lack of investment in long-term care services, including digital solutions, and of alignment with public health policies, were other constraints discussed.

All the patients and caregivers revealed interest with this remote physician–patient interaction and asked for extension of the “BALANCE” digital service use. The motivation for physical activity is a well-defined public health policy for healthy ageing [43,44] and with the “BALANCE” digital service, we are motivating this in a clinical way.

All the physicians also recognized the potential of the “BALANCE” digital service to be used as a complementary healthcare provision, once the interoperability and the restructuring of work processes are guaranteed [41].

Face-to-face physician–patient interaction will always be important. However, several researchers have observed a gradual path for the medical specialties to learn how to reach their patients remotely and benefit from it [45,46,47].

Our study allowed us to design and develop, using a scientifically-based approach, the “BALANCE” digital service, a digital supporting system that could add value for complementary treatment, and optimize and maintain opportunities for elderly people with functional ability within their communities. Again, we can also consider that these systems could support the training and preparing of caregivers for their roles, promoting active ageing and proactively using a digital solution. There is always an additional effort to understand and implement innovative technologies, but it is necessary to establish implementation strategies to overcome this challenge.

### Limitations

Regarding the eligible health professionals, this project only included physicians. In future studies, we must consider the participation of other professionals with healthcare provision for the elderly with balance disorders and risk of falling. The sampling of the all those interviewed was intentional, and the focus groups were conducted remotely via Zoom due to SARS-CoV-2 pandemic limitations.

Relative to the patients, we included participants without diagnosed neurologic disease. In fact, patients with balance disorders and neurologic diseases could benefit from the “BALANCE” digital service. However, future researchers could evaluate whether constraints with the use of this digital solution by neurological patients would be greater.

Because we performed a proof-of-concept study, only one physician (the researcher) and five patients, followed up at HBA and participated in the demonstration of the digital solution. Additionally, the follow-up time was only three months. However, after this initial intervention, we are planning to carry out a future study with a longer follow-up and a representative sample, considering the suggestions of the focus groups.

Finally, only the participants of the proof-of-concept study and the invited physicians evaluated the “BALANCE” digital service. In fact, managers and digital developers must also be involved in the evaluation of digital health solutions [33,48].

Multicenter studies with a longer follow-up could provide more information on usability, acceptability, adherence and impact of the “BALANCE” digital service.

## 5. Conclusions

Using DSRM, we designed, implemented and demonstrated “BALANCE”, a digital service for complementary care provision for elderly people with balance disorders and risk of falling. We explored the benefits, constraints, necessary adjustments and satisfaction. We verified a significant potential for clinical applicability of this digital solution. However, involvement of patients, caregivers and healthcare professionals, interoperability of digital solutions and reorganization of work activities are key points for future clinical applicability. Additional studies are required to include these aspects.

## Figures and Tables

**Table 1:** Socio-demographic data of the participants in the proof-of-concept.

Patient	Patient Gender	Patient Age	Education Level (Years of Schooling)	MMSE * Score	Caregiver Gender	Caregiver Age	Caregiver Education Level (Years of Schooling)
1	F	70	6	30	F	72	4
2	F	71	+7	28	-	-	-
3	F	80	6	27	F	58	+7
4	F	83	4	24	F/F	38/59	+7/+7
5	F	83	+7	30	F	53	+7

* MMSE: Mini-Mental State Examination.

**Table 2:** Patient and caregiver registration.

Patient	Complaints of Difficulty with Exercise Performance (Platform Message)	Complains of Clinical Deterioration (Telephone or Zoom)	Record of Clinical Deterioration on the Platform	Complains of Falls (Telephone or Zoom)	Record of Falls on the Platform	DHI Score Pre Rehabilitation	DHI Score Post Rehabilitation	Record of Balance Exercises Performed	Upload of Video with Exercises Performed
1	Sometimes	Sometimes	Not filled	No	Not filled	Not filled	Not filled	Not filled	No
2	No	Once	Once	No	Not filled	24	13	Not filled	No
3	Several times	No	Not filled	No	Not filled	68	56	Incomplete 27 days	No
4	No	Once	Not filled	No	Not filled	28	16	Not filled	No
5	Several times	Once	Once	No	Not filled	90	Not filled	Incomplete 20 days	No

**Table 3:** Socio-demographic data of the physicians.

Physician	Gender	Age	Specialty	Regional Health Administration of Portugal
1	M	49	Family Medicine	LTV
2	F	68	Family Medicine	Center
3	M	59	Internal Medicine	LTV
4	F	60	Internal Medicine	LTV
5	M	58	Otorhinolaryngology	LTV
6	M	59	Otorhinolaryngology	North
7	M	61	Otorhinolaryngology	LTV
8	M	68	Otorhinolaryngology	Center

M: Male; F: Female; LTV: Lisbon and Tejo Valey.

**Table 4:** Thematic categories of the focus groups.

Thematic Categories	Focus Group: Patients and Caregivers	Focus Group: Physicians
1. Benefits of “BALANCE” digital service	1.1. Patient comfort 1.2. Closer physician-patient interaction anywhere 1.3. Availability of tutorial videos with balance exercises	1.1. Patient comfort 1.2. Closer physician-patient interaction 1.3. Lower consumption of face-to-face resources 1.4. Patient motivation and adhesion
2. Constraints regarding the use of “BALANCE” digital service	2.1. Patient profile 2.2. Presentation screen on the mobile phone 2.3. Lack of time to record the exercises performed	2.1. Patient profile 2.2. Resistance of healthcare professionals 2.3. Lack of working time by healthcare professionals
3. Satisfaction with “BALANCE” digital service functionalities	3.1. Satisfaction level	3.1. Satisfaction level
4. Suggested strategies to improve “BALANCE” digital service	4.1. Presentation screen of “BALANCE” 4.2. Recorded videos with more identifiable exercises 4.3. Adjustment of interactive data relatively to performed balance exercises	4.1. Inclusion of automatic tools—objective data of performed balance exercises
5. Suggested strategies for new clinical applicability of “BALANCE” digital service	-	5.1. Working hours organization 5.2. Involvement of other health professionals 5.3. Interoperability 5.4. Funding and continuity of the use of digital solution 5.5. Recognition of the hierarchy
6. Interest in using “BALANCE”digital service	6.1. Interest in maintaining the use	6.1. Recognized interest in using “BALANCE” digital service

## Data Availability

The data that support the findings of this study are available on request from the corresponding author A.G.M.G.

## Appendix A

**Table A1:** Focus group with patients and caregivers: Interview guide.

Thematic Categories	Questions
Benefits of “BALANCE” service	“In your opinion, was this digital service beneficial? Why?”
Constraints relatively the digital service	“Did you have difficulty using this digital service?” If yes, what were the difficulties“
Satisfaction	“What is your level of satisfaction regarding the use of the “BALANCE” service?”
Strategies to improve the digital service	“What would you recommend to improve this service?”
Interest in maintaining the use	“Would you like to continue using the “BALANCE” service?”

**Table A2:** Focus group with physicians: Interview guide.

Thematic Categories	Questions
Benefits of “BALANCE” service	“What do you think about the benefits of this digital service for the provision of complementary healthcare for elderly people with balance disorders and risk of falling?”
Constraints regarding the use of “BALANCE” service	“What is your opinion about the constrains/limitations regarding this service?”
Medical satisfaction with “BALANCE” service functionalities	“How satisfied are you with the potential of this digital service?”
Suggested strategies to adjust “BALANCE” service	“What strategies can be implemented to improve BALANCE service?”
Suggested strategies for clinical applicability	“How can “BALANCE” service be suitable for clinical applicability?”
Interest in the “BALANCE” service	“In your opinion, is there interest in this digital complementary health service?”

## References

[1] World Health Organization (WHO) (2020). Regional Office for Europe. Portugal-Country Case Study on the Integrated Delivery of Long-Term Care. https://www.euro.who.int/__data/assets/pdf_file/0004/426388/05_PORT-CountryRep_WEB.pdf.

[2] WHO Regional Office for Europe (2017). Age-Friendly Environments in Europe. A Handbook of Domains for Policy Action. https://apps.who.int/iris/bitstream/handle/10665/334251/9789289052887-eng.pdf.

[3] World Health Organization (WHO) (2020). Decade of Healthy Ageing. Baseline Report. https://www.who.int/publications/i/item/9789240017900.

[4] Rudnicka E., Napierała P., Podfigurna A., Męczekalski B., Smolarczyk R., Grymowicz M. (2020). The World Health Organization (WHO) approach to healthy ageing. Maturitas.

[5] OECD (2021). Health at a Glance 2021: OECD Indicators.

[6] Fernández L., Breinbauer H.A., Delano P.H. (2015). Vertigo and Dizziness in the Elderly. Front. Neurol..

[7] Nguyen H., Moreno-Agostino D., Chua K.C., Vitoratou S., Prina A.M. (2021). Trajectories of healthy ageing among older adults with multimorbidity: A growth mixture model using harmonised data from eight ATHLOS cohorts. PLoS ONE.

[8] Casani A.P., Gufoni M., Capobianco S. (2021). Current Insights into Treating Vertigo in Older Adults. Drugs Aging.

[9] Ha V.A.T., Nguyen T.N., Nguyen T.X., Nguyen H.T.T., Nguyen T.T.H., Nguyen A.T., Pham T., Thanh Vu H.T. (2021). Prevalence and Factors Associated with Falls among Older Outpatients. Int. J. Environ. Res. Public Health.

[10] Coto J., Alvarez C.L., Cejas I., Colbert B.M., Levin B.E., Huppert J., Rundek T., Balaban C., Blanton S.H., Lee D.J. (2021). Peripheral vestibular system: Age-related vestibular loss and associated deficits. J. Otol..

[11] Zhang R., Liu B., Bi J., Chen Y. (2021). Relationship Between Chronic Conditions and Balance Disorders in Outpatients with Dizziness: A Hospital-Based Cross-Sectional Study. Med. Sci. Monit..

[12] Florence C.S., Bergen G., Atherly A., Burns E., Stevens J., Drake C. (2018). Medical Costs of Fatal and Nonfatal Falls in Older Adults. J. Am. Geriatr. Soc..

[13] Kovacs E., Wang X., Grill E. (2019). Economic burden of vertigo: A systematic review. Health Econ. Rev..

[14] Lapão L.V., Dussault G. (2017). The contribution of eHealth and mHealth to improving the performance of the health workforce: A review. Public Health Panor..

[15] Uei S.L., Kuo Y.M., Tsai C.H., Kuo Y.L. (2017). An Exploration of Intent to Use Telehealth at Home for Patients with Chronic Diseases. Int. J. Environ. Res. Public Health.

[16] Olsen C.F., Bergland A., Bye A., Debesay J., Langaas A.G. (2021). Crossing knowledge boundaries: Health care providers’ perceptions and experiences of what is important to achieve more person-centered patient pathways for older people. BMC Health Serv. Res..

[17] Wynn R., Gabarron E., Johnsen J.K., Traver V. (2020). Special Issue on E-Health Services. Int. J. Environ. Res. Public Health.

[18] Mucchi L., Jayousi S., Gant A., Paoletti E., Zoppi P. (2021). Tele-Monitoring System for Chronic Diseases Management: Requirements and Architecture. Int. J. Environ. Res. Public Health.

[19] Leirós-Rodríguez R., García-Soidán J.L., Romo-Pérez V. (2019). Analyzing the Use of Accelerometers as a Method of Early Diagnosis of Alterations in Balance in Elderly People: A Systematic Review. Sensors.

[20] Choi S.D., Guo L., Kang D., Xiong S. (2017). Exergame technology and interactive interventions for elderly fall prevention: A systematic literature review. Appl. Ergon..

[21] Gaspar A.G.M., Lapão L.V. (2021). eHealth for Addressing Balance Disorders in the Elderly: Systematic Review. J. Med. Internet Res..

[22] Peffers K., Tuunanen T., Rothenberger M.A., Chatterjee S. (2007). A design science research methodology for information systems research. J. Manag. Inf. Syst..

[23] Gaspar A.G.M., Escada P., Lapão L.V. (2021). How Can We Develop an Efficient eHealth Service for Provision of Care for Elderly People with Balance Disorders and Risk of Falling? A Mixed Methods Study. Int. J. Environ. Res. Public Health.

[24] Brandt T., Dieterich M., Strupp M. (2014). Vertigo and Dizziness: Common Complaints.

[25] Jacobson G.P., Newman C.W. (1990). The development of the Dizziness Handicap Inventory. Arch. Otolaryngol. Head Neck Surg..

[26] Lapão L.V., Peyroteo M., Maia M., Seixas J., Gregório J., Mira da Silva M., Heleno B., Correia J.C. (2021). Implementation of Digital Monitoring Services During the COVID-19 Pandemic for Patients with Chronic Diseases: Design Science Approach. J. Med. Internet Res..

[27] Blokdyk G. (2021). Proof of Concept Poc. A Complete Guide-2020.

[28] Morgado J., Rocha C.S., Maruta C., Guerreiro M., Martins I.P. (2009). Novos valores normativos do Mini-Mental State Examination. Sinapse.

[29] Creswell J.W., Creswell J.D. (2018). Research design. Qualitative, Quantitative, and Mixed Methods Approaches.

[30] Assembleia da República Lei nº 58/2019 de 8 de Agosto de 2019. Diário da República n.º 151/2019, Série I de 2019-08-08. 3–40. https://data.dre.pt/eli/lei/58/2019/08/08/p/dre.

[31] General Data Protection Regulation Art. 12 GDPR–Transparent Information, Communication and Modalities for the Exercise of the Rights of the Data Subject-General Data Protection Regulation (GDPR). Gdpr-info.eu.

[32] Pearce P.F., Ferguson L.A., George G.S., Langford C.A. (2016). The essential SOAP note in an EHR age. Nurse Pract..

[33] Goldsack J.C., Zanetti C.A. (2020). Defining and Developing the Workforce Needed for Success in the Digital Era of Medicine. Digit. Biomark..

[34] Tossaint-Schoenmakers R., Versluis A., Chavannes N., Talboom-Kamp E., Kasteleyn M. (2021). The Challenge of Integrating eHealth Into Health Care: Systematic Literature Review of the Donabedian Model of Structure, Process, and Outcome. J. Med. Internet Res..

[35] Kim H.S., Kwon I.H., Cha W.C. (2021). Future and Development Direction of Digital Healthcare. Healthc. Inform. Res..

[36] Van Vugt V.A., van der Wouden J.C., Essery R., Yardley L., Twisk J.W., van der Horst H.E., Maarsingh O.R. (2019). Internet based vestibular rehabilitation with and without physiotherapy support for adults aged 50 and older with a chronic vestibular syndrome in general practice: Three armed randomised controlled trial. BMJ.

[37] Orton M., Agarwal S., Muhoza P., Vasudevan L., Vu A. (2018). Strengthening Delivery of Health Services Using Digital Devices. Glob. Health Sci. Pract..

[38] Ricciardi W., Pita Barros P., Bourek A., Brouwer W., Kelsey T., Lehtonen L. (2019). How to govern the digital transformation of health services. Eur. J. Public Health.

[39] Jun W. (2020). A Study on the Current Status and Improvement of the Digital Divide among Older People in Korea. Int. J. Environ. Res. Public Health.

[40] Heponiemi T., Jormanainen V., Leemann L., Manderbacka K., Aalto A.M., Hyppönen H. (2020). Digital Divide in Perceived Benefits of Online Health Care and Social Welfare Services: National Cross-Sectional Survey Study. J. Med. Internet Res..

[41] Trenerry B., Chng S., Wang Y., Suhaila Z.S., Lim S.S., Lu H.Y., Oh P.H. (2021). Preparing Workplaces for Digital Transformation: An Integrative Review and Framework of Multi-Level Factors. Front. Psychol..

[42] Raja M., Bjerkan J., Kymre I.G., Galvin K.T., Uhrenfeldt L. (2021). Telehealth and digital developments in society that persons 75 years and older in European countries have been part of: A scoping review. BMC Health Serv. Res..

[43] Sowa A., Tobiasz-Adamczyk B., Topór-Mądry R., Poscia A., la Milia D.I. (2016). Predictors of healthy ageing: Public health policy targets. BMC Health Serv. Res..

[44] Bechtold U., Stauder N., Fieder M. (2021). Let’s Walk It: Mobility and the Perceived Quality of Life in Older Adults. Int. J. Environ. Res. Public Health.

[45] Cohen A.B., Nahed B.V. (2021). The Digital Neurologic Examination. Digit. Biomark..

[46] Barreto R.G., Yacovino D.A., Cherchi M., Nader S.N., Teixeira L.J., Silva D.A.D., Verdecchia D.H. (2021). The Role of the Smartphone in the Diagnosis of Vestibular Hypofunction: A Clinical Strategy for Teleconsultation during the COVID-19 Pandemic and Beyond. Int. Arch. Otorhinolaryngol..

[47] Da Fonseca M.H., Kovaleski F., Picinin C.T., Pedroso B., Rubbo P. (2021). E-Health Practices and Technologies: A Systematic Review from 2014 to 2019. Healthcare.

[48] Karpathakis K., Libow G., Potts H.W.W., Dixon S., Greaves F., Murray E. (2021). An Evaluation Service for Digital Public Health Interventions: User-Centered Design Approach. J. Med. Internet Res..

